# Estrogen Modulates Specific Life and Death Signals Induced by LH and hCG in Human Primary Granulosa Cells In Vitro

**DOI:** 10.3390/ijms18050926

**Published:** 2017-04-28

**Authors:** Livio Casarini, Laura Riccetti, Francesco De Pascali, Lisa Gilioli, Marco Marino, Eugenia Vecchi, Daria Morini, Alessia Nicoli, Giovanni Battista La Sala, Manuela Simoni

**Affiliations:** 1 Unit of Endocrinology, Department of Biomedical, Metabolic and Neural Sciences, University of Modena and Reggio Emilia, NOCSAE, via P. Giardini 1355, 41126 Modena, Italy; laura.riccetti@unimore.it (L.R.); francescodepascali@outlook.com (F.D.P.); 163088@studenti.unimore.it (L.G.); marco.marino@unimore.it (M.M.); eugenia.vecchi@hotmail.it (E.V.); manuela.simoni@unimore.it (M.S.); 2 Center for Genomic Research, University of Modena and Reggio Emilia, Via G. Campi 287, 41125 Modena, Italy; 3 Department of Medicine, Endocrinology, Metabolism and Geriatrics, Azienda USL di Modena, 41124 Modena, Italy; 4 Units of Obstetrics and Gynecology, IRCCS-Arcispedale Santa Maria Nuova, Via Risorgimento 80, 42123 Reggio Emilia, Italy; Daria.Morini@asmn.re.it (D.M.); Alessia.Nicoli@asmn.re.it (A.N.); GiovanniBattista.LaSala@asmn.re.it (G.B.L.S.); 5 Department of Medical and Surgical Sciences for Children and Adults, University of Modena and Reggio Emilia, Via del Pozzo 71, 41124 Modena, Italy

**Keywords:** LH, hCG, gonadotropins, apoptosis, granulosa

## Abstract

Luteinizing hormone (LH) and human chorionic gonadotropin (hCG) are glycoprotein hormones used for assisted reproduction acting on the same receptor (LHCGR) and mediating different intracellular signaling. We evaluated the pro- and anti-apoptotic effect of 100 pM LH or hCG, in the presence or in the absence of 200 pg/mL 17β-estradiol, in long-term, serum-starved human primary granulosa cells (hGLC) and a transfected granulosa cell line overexpressing LHCGR (hGL5/LHCGR). To this purpose, phospho-extracellular-regulated kinase 1/2 (pERK1/2), protein kinase B (pAKT), cAMP-responsive element binding protein (pCREB) activation and procaspase 3 cleavage were evaluated over three days by Western blotting, along with the expression of target genes by real-time PCR and cell viability by colorimetric assay. We found that LH induced predominant pERK1/2 and pAKT activation *STARD1*, *CCND2* and anti-apoptotic *XIAP* gene expression, while hCG mediated more potent CREB phosphorylation, expression of *CYP19A1* and procaspase 3 cleavage than LH. Cell treatment by LH is accompanied by increased (serum-starved) cell viability, while hCG decreased the number of viable cells. The hCG-specific, pro-apoptotic effect was blocked by a physiological dose of 17β-estradiol, resulting in pAKT activation, lack of procaspase 3 cleavage and increased cell viability. These results confirm that relatively high levels of steroidogenic pathway activation are linked to pro-apoptotic signals in vitro, which may be counteracted by other factors, i.e., estrogens.

## 1. Introduction

Gonadotropins are glycoprotein hormones regulating development and reproduction. In women of fertile age, they regulate menstrual cycle, oocyte growth, ovulation and pregnancy. During follicular maturation life and death signals occur for selection of the dominant follicle, which undergo full maturation, while the others become atretic via apoptotic processes. Follicle-stimulating hormone (FSH) induces the growth and maturation of follicles and estrogens synthesis, in concert with the luteinizing hormone (LH), until ovulation. A third gonadotropin, i.e., chorionic gonadotropin (CG) exists in primates to induce progesterone production and support pregnancy [1,2,3].

### 1.1. Gonadotropin Receptors-Mediated Signals

Gonadotropins act by binding to their G protein-coupled receptors (GPCRs), which share similar gene sequences and protein structure. While FSH is the unique ligand for its receptor (FSHR), LH and hCG bind a common receptor (LHCGR) expressed in the gonads [2,3] and are used since two decades ago as an equivalent supplementation to FSH for controlled ovarian stimulation in assisted reproduction techniques (ART). The LHCGR is encoded by a gene spanning about 20 Kbps featured by 11 exons: exons 1–10 encode the extracellular domain, comprising part of the hinge region, while the rest of the hinge region, the seven transmembrane domains with intra- and extra-cellular loops, and the C-terminal portion of the receptor are encoded by exon 10–11. An additional, primate-specific exon (6A) transcribed as a spicing variant and producing several *LHCGR* mRNA isoforms was found [4], suggesting that further levels of regulation of the receptor activity may exist.

Upon hormone binding, the tertiary structure of LHCGR undergoes conformational changes, inducing the simultaneous activation of multiple intracellular signaling cascades in granulosa cells. The Gαs/cAMP/protein kinase A (PKA)-pathway is classically known to be associated to steroidogenic events and is characterized by the activation of the adenylyl cyclase enzyme, which results in intracellular cAMP increase, PKA activation, extracellular-regulated kinase (ERK1/2) and cAMP-responsive element binding protein (CREB) phosphorylation and transcription of target genes [2]. A number of these genes encode for enzymes such as steroidogenic acute regulatory protein (StAR), which regulates the transport of cholesterol within the mitochondria, and aromatase, a catalyzer of androgens conversion to estrogens [5]. Besides the steroidogenic effect, ERK1/2 phosphorylation, which may occur via alternative molecules (e.g., β-arrestins) [6], is linked to GPCR internalization [7] and granulosa cell proliferation [8], as well as the counterbalancing of steroidogenic and pro-apoptotic signals [9]. Indeed, cell death may occur in concomitance with steroidogenic cAMP/PKA-pathway activation, inducing cell rounding and p53 activation via cAMP-dependent cross-talk between intracellular signaling pathways [9,10,11,12,13]. LHCGR-mediated anti-apoptotic effects are exerted via PI3K/AKT-pathway, which is upregulated by LH treatment in cultured granulosa cells [14,15,16,17].

### 1.2. Differences between Luteinizing Hormone (LH) and Human Chorionic Gonadotropin (hCG) at the Molecular Levels

The LHCGR is capable of discriminating between the ligands and modulates qualitatively different, LH- and hCG-specific intracellular signaling [14,16,18]. In cultured human granulosa cells, dose-response experiments revealed about a five-fold lower effective dose 50% (EC_50_) of hCG than LH, in inducing cAMP production [14], while LH treatment induced prevalent pERK1/2 and pAKT activation, which was potentiated in the presence of FSH [16]. These in vitro studies focused on the characterization of ligand-dependent signals, mediated by LHCGR, using increasing gonadotropin dosages falling within the pM–nM range. The activation of pERK1/2, pAKT and pCREB, as well as cAMP, were detected by Western blotting experiments and enzyme-linked immunosorbent assay (ELISA), respectively, revealing gonadotropin specific signaling and in vitro potency. These ligand-specific effects seem to be peculiarly mediated by the human LHCGR, since the rodent receptor is capable only of quantitative, but not qualitative discrimination of LH- and hCG-specific signaling [18]. Anyway, in vitro data may reflect the physiological role of the two gonadotropins. LH is produced by the pituitary to modulate dominant follicle growth and maturation, reasonably via activation of proliferative and anti-apoptotic signals, while hCG is the pregnancy hormone, which drives embryo development by massive progesterone synthesis.

### 1.3. Role of Estrogens in the Ovary

A number of molecules contribute in modulating intracellular signaling in the ovary [19,20]. Estrogen synthesis accompanies the regulatory functions of gonadotropins in the ovary exerting proliferative and differentiation actions on the ovarian follicle [21,22]. Estrogens, predominantly 17β-estradiol, act by binding to two nuclear receptors (ERα and ERβ) and a recently discovered GPCR (GPER), all expressed in granulosa cells [23]. Since these receptors are positive modulators of ERK1/2- and AKT-pathways, the action of estrogens was linked to cell growth and tumor progression in several cell models [24,25,26,27], and it is reasonable that these hormones may play a role in the regulation of gonadotropin-mediated intracellular signaling.

### 1.4. Aim of the Study

This study was undertaken to demonstrate how LH- and hCG-specific intracellular signaling may be linked to downstream life and death signals in human primary granulosa lutein cells (hGLC) in vitro. Moreover, the role of 17β-estradiol, reasonably acting simultaneously with gonadotropins in vivo, was evaluated. To this purpose, cultured, serum-starved hGLC were exposed to LH and hCG over three days and the activation of phospho-proteins, gene expression, procaspase 3 cleavage and cell viability were evaluated. Results were confirmed using an immortalized human granulosa cell line over-expressing LHCGR. The investigation of specific LH- and hCG-mediated signaling pathways and downstream life and death signals activated by the common receptor, as well as the modulatory role of estrogens on granulosa cells, provide useful information for ART.

## 2. Results

### 2.1. Cell Signaling Analysis and Procaspase 3 Cleavage

**Figure 1:**
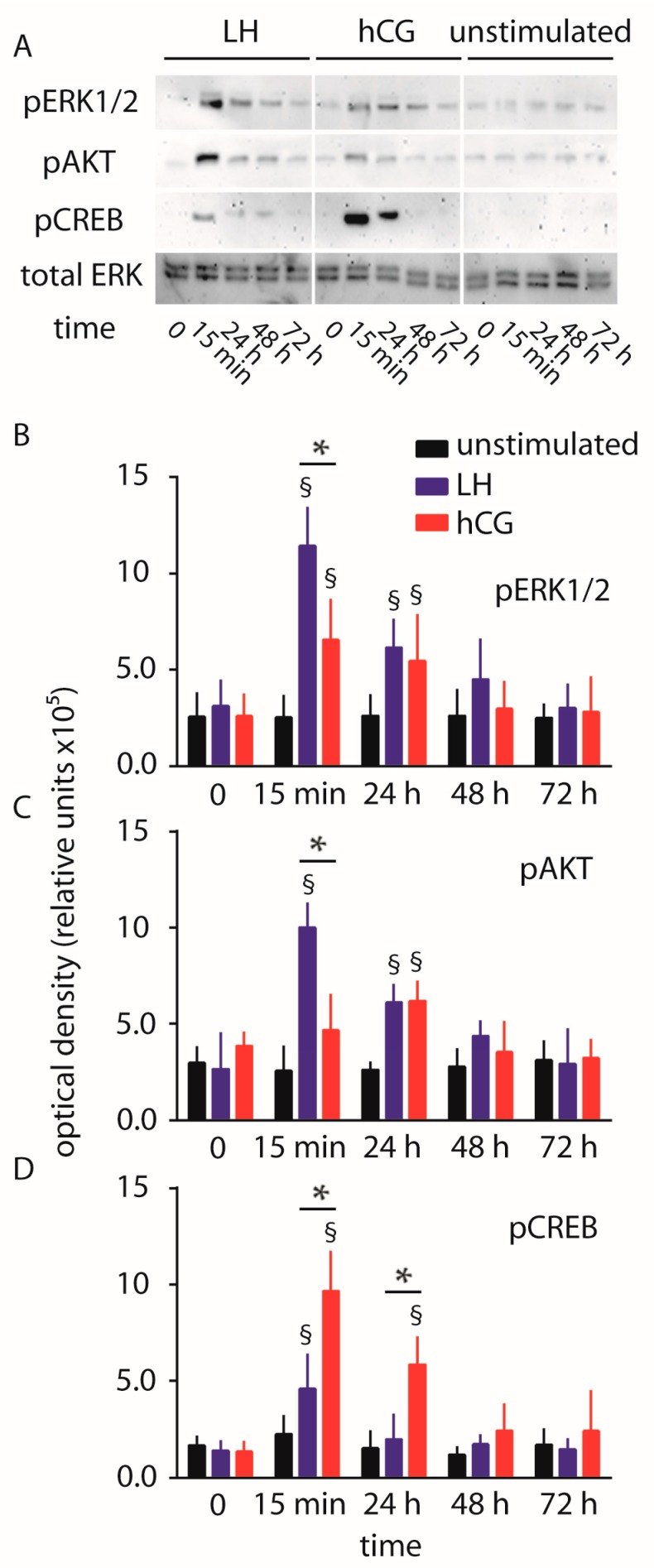
Comparison of luteinizing hormone (LH)- and human chorionic gonadotropin (hCG)-induced extracellular-regulated kinase 1/2 (ERK1/2), protein kinase B (AKT) and cAMP-responsive element binding protein (CREB) phosphorylation over 72 h, in human primary granulosa lutein cells (hGLC). (**A**) Evaluation of pERK1/2, pAKT and pCREB activation by Western blotting. Total ERK served as loading control (images representative of four independent experiments); (**B**–**D**) Semi-quantification of pERK1/2, pAKT and pCREB Western blotting signals. § = significantly different to unstimulated (control) at the same time-point; * = significant difference of LH versus hCG; two-way ANOVA and Bonferroni post-test (*p* < 0.05; means ± standard deviation (SD); *n* = 4).

hGLC were maintained under continuous treatment with 100 pM LH or hCG, as a gonadotropin dose maximally activating phospho-proteins in hGLC over three days [9,14,16,28]. The phosphorylation of ERK1/2, AKT and CREB was evaluated by Western blotting, together with procaspase 3 cleavage. Protein signals were semi-quantified by an analysis software and represented in graphs (Figure 1).

We confirmed that 15 min of 100 pM LH treatment induces higher levels of both pERK1/2 and pAKT activation than 100 pM hCG (two-way ANOVA; *p* < 0.05; *n* = 4), as described previously [14]. Moreover, the LH and hCG-induced phosphorylation of AKT is significantly higher than basal level at the 24 h time-point. Interestingly, hCG treatment of hGLC results in higher levels of pCREB activation than LH within 15 min–24 h (two-way ANOVA; *p* < 0.05; *n* = 4). These results were confirmed using hGL5/LHCGR cell line, where time-course experiments were extended until five days due to higher viability of this cell model compared to hGLC (Figure S1).

**Figure 2:**
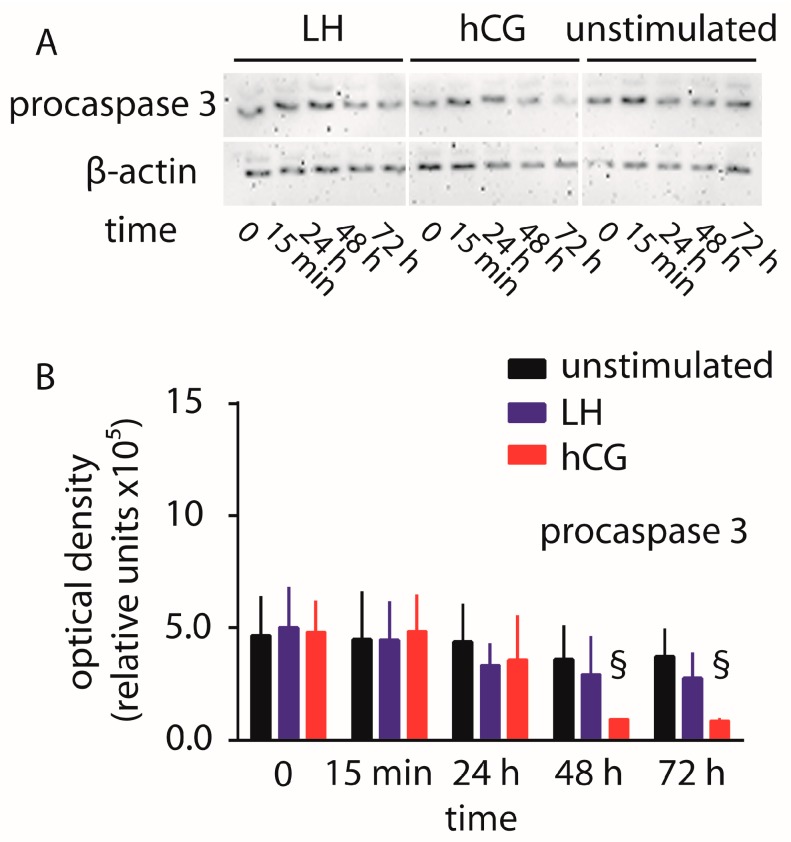
Evaluation of LH- and hCG-induced procaspase 3 cleavage over 72 h, in hGLC. (**A**) Evaluation of procaspase 3 by Western blotting. β-actin was the loading control (images representative of four independent experiments); (**B**) Semi-quantification of procaspase 3 Western blotting signals. § = significantly different versus unstimulated (control) samples collected at the same time-point; two-way ANOVA and Bonferroni post-test (*p* < 0.05; means ± SD; *n* = 4).

The amount of uncleaved procaspase 3 was evaluated in hGLC maintained under 100 pM LH or hCG over 72 h (Figure 2). While no significant changes in procaspase 3 levels were detected in cells treated by LH or untreated cells, the continuous exposure of hGLC to hCG resulted in signal decay within 2–3 days, compared to controls, indicating the procaspase 3 cleavage as a pro-apoptotic event.

### 2.2. Gene Expression Analysis

**Figure 3:**
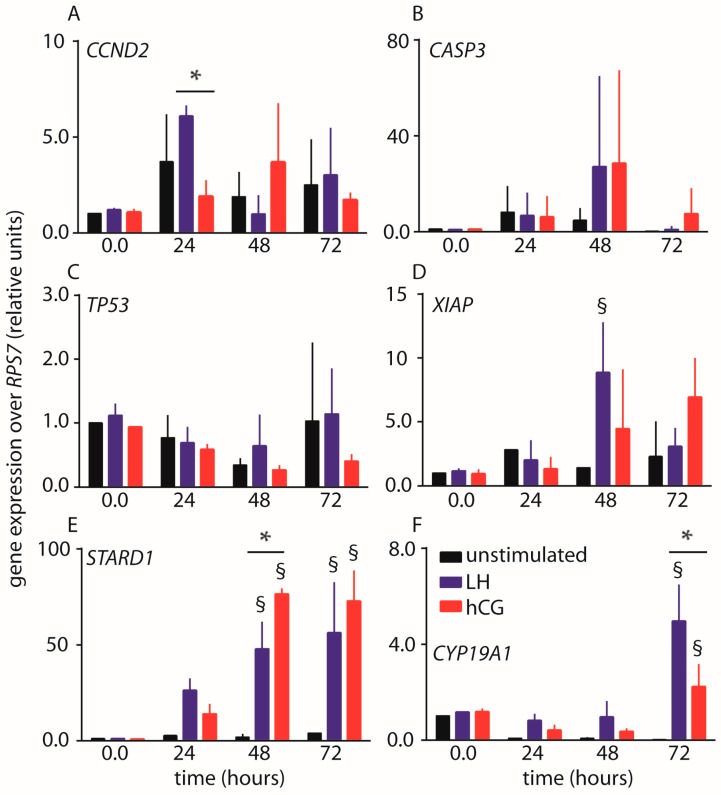
Time-course (0–72 h) expression analysis of LH- and hCG-target genes by real-time PCR. Values were normalized over the expression of *RPS7* housekeeping gene. (**A**) *CCND2* gene encoding for the cyclin D2; (**B**) *CASP3* gene encoding for the procaspase 3; (**C**) *TP53* gene encoding for the p53 tumor-suppressor protein; (**D**) *XIAP* gene encoding for the X-linked inhibitor of apoptosis factor; (**E**) *STARD1* gene encoding for the StAR enzyme; (**F**) *CYP19A1* gene encoding for the aromatase enzyme. § = significantly different to unstimulated (control) at the same time-point; * = significant difference of LH versus hCG; two-way ANOVA and Bonferroni post-test (*p* < 0.05; means ± SD; *n* = 3).

The expression of genes regulating cell cycle, pro- or anti-apoptotic signals, and steroidogenesis was evaluated by real-time PCR in hGLC maintained under continuous 100 pM LH or hCG stimulation. Data collected over 72 h were graphically represented as means ± standard deviation (SD) (Figure 3).

The expression of *CCND2* gene encoding for the cell cycle regulator cycline D2 is variable over time, resulting in increased mRNA levels upon LH stimulation within 24 h (two-way ANOVA; *p* < 0.05; *n* = 3). Induction of *CCND2* gene expression peak is delayed by hCG treatment occurring at about 48 h, although it does not significantly differ to control, even though both gonadotropins result in *CCND2* transcription levels similar to those of controls within 72 h.

*CASP3* gene encodes for the procaspase 3 enzyme and it is not sufficiently upregulated upon treatment by either hormone, resulting in no statistically different levels compared to controls (two-way ANOVA; *p* ≥ 0.05; *n* = 3). However, marked increase of *CASP3* gene expression was revealed upon 100 pM hCG treatment of the hGL5/LHCGR cell line over five days (Figure S2). In hGLC, the expression of pro-apoptotic *TP53* gene is not upregulated by 100 pM LH or hCG over 72 h (two-way ANOVA; *p* ≥ 0.05; *n* = 3). Cell treatment by LH and hCG results in different *XIAP* gene expression, which is an inhibitor of apoptosis. Especially, LH-induced *XIAP* gene expression achieved the maximal levels within 48 h (two-way ANOVA; *p* < 0.05; *n* = 3), while no statistically differences between hCG-treated cells and unstimulated controls were found (Figure 3).

Finally, we found that both the treatments by LH and hCG of hGLC results in increased *STARD1* and *CYP19A1* gene expression at 48–72 and 72 h, respectively (two-way ANOVA; *p* < 0.05; *n* = 3), encoding for key regulators of steroidogenesis, such as the steroidogenic acute regulatory protein and aromatase enzymes. In particular, *STARD1* upregulation is more pronounced upon hCG than LH treatment, which, in turn, induced higher levels of *CYP19A1* gene expression (Figure 3).

Taken together, specific LH- and hCG-mediated gene expression is not detected at all the time points considered and in all the genes examined. However, prevalent LH proliferative and anti-apoptotic actions were revealed by *CCND2* and *XIAP* gene expression increase, compared to hCG. Moreover, the hormones induced differently *STARD1* and *CYP19A1* gene expression, suggesting that the modulation of steroidogenic signals is ligand-specific and may occur at different levels.

### 2.3. Evaluation of Cell Viability

**Figure 4:**
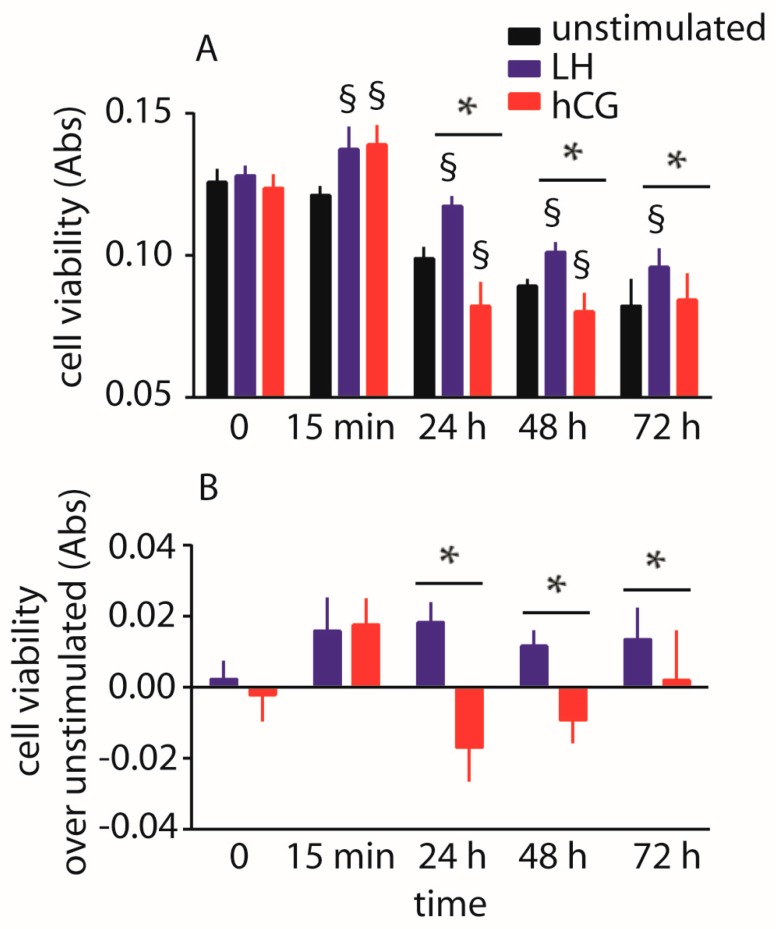
Analysis of LH- and hCG-treated (0–72 h), serum-starved hGLC viability by 3-(4,5-dimethylthiazol-2-yl)-2,5-diphenyltetrazolium bromide (MTT) assay. Cells maintained in the absence of gonadotropin served as controls. (**A**) Directly measured absorbance values; (**B**) Cell viability values expressed as increase or decrease over basal levels. § = significantly different to unstimulated (control) at the same time-point; * = significant difference of LH versus hCG; two-way ANOVA and Bonferroni post-test (*p* < 0.05; means ± SD; *n* = 10).

The impact of 100 pM LH and hCG on hGLC viability maintained under continuous exposure to gonadotropins was evaluated over three days by 3-(4,5-dimethylthiazol-2-yl)-2,5-diphenyltetrazolium bromide (MTT) assay. The experiments were performed in the absence of serum to avoid background disturbance and, especially, to induce pro-apoptotic signals [16]. Data were collected over 72 h and represented as means ± SD in a graph (Figure 4).

We found a positive effect on viability of cells maintained under LH exposure over 72 h, while the treatment by hCG decreases cell viability (two-way ANOVA; *p* < 0.05; *n* = 10). These effects mediated by gonadotropins are assessable in the 24–48 h time range, while an overall decrease of cell viability is anyway observed in all the samples over 72 h, when the levels of absorbance achieve values one-third lower than those measured at the first time-point (*t*-test; *p* < 0.05; *n* = 10). This experiment was repeated using the hGL5/LHCGR cell line and similar results were obtained (Figure S3).

### 2.4. Abolition of Gonadotropin-Mediated Pro-Apoptotic Signals by Estrogens

Study of intracellular mechanisms take advantage from in vitro systems, where modulating effects of cell metabolism and molecular cross-interaction with additional factors are limited, if not absent. This allows to characterize the effect of single compounds at the molecular level, which, however, may not be easily detectable in vivo due to the complexity of the background and the concomitance of several, putatively opposite signals. Therefore, at odds with the available in vitro evidence, the use of LH or hCG supplementation is apparently equally effective in FSH-induced multi-follicular growth in assisted reproduction. In this case, both LH and hCG treatments support granulosa cell growth and oocyte maturation, reasonably through upregulation of proliferative signals.

To assess whether hCG-dependent pro-apoptotic stimuli may be modulated, hGLC were maintained under 100 pM LH or hCG exposure over 72 h, in the presence of 200 pg/mL 17β-estradiol (~730 pM). Estrogens are synthesized by ovarian follicles upon conversion of androgens and mediate proliferative signals [29]. The phosphorylation of AKT, procaspase 3 cleavage and cell viability were evaluated after the various treatments by Western blotting and MTT assay, respectively.

**Figure 5:**
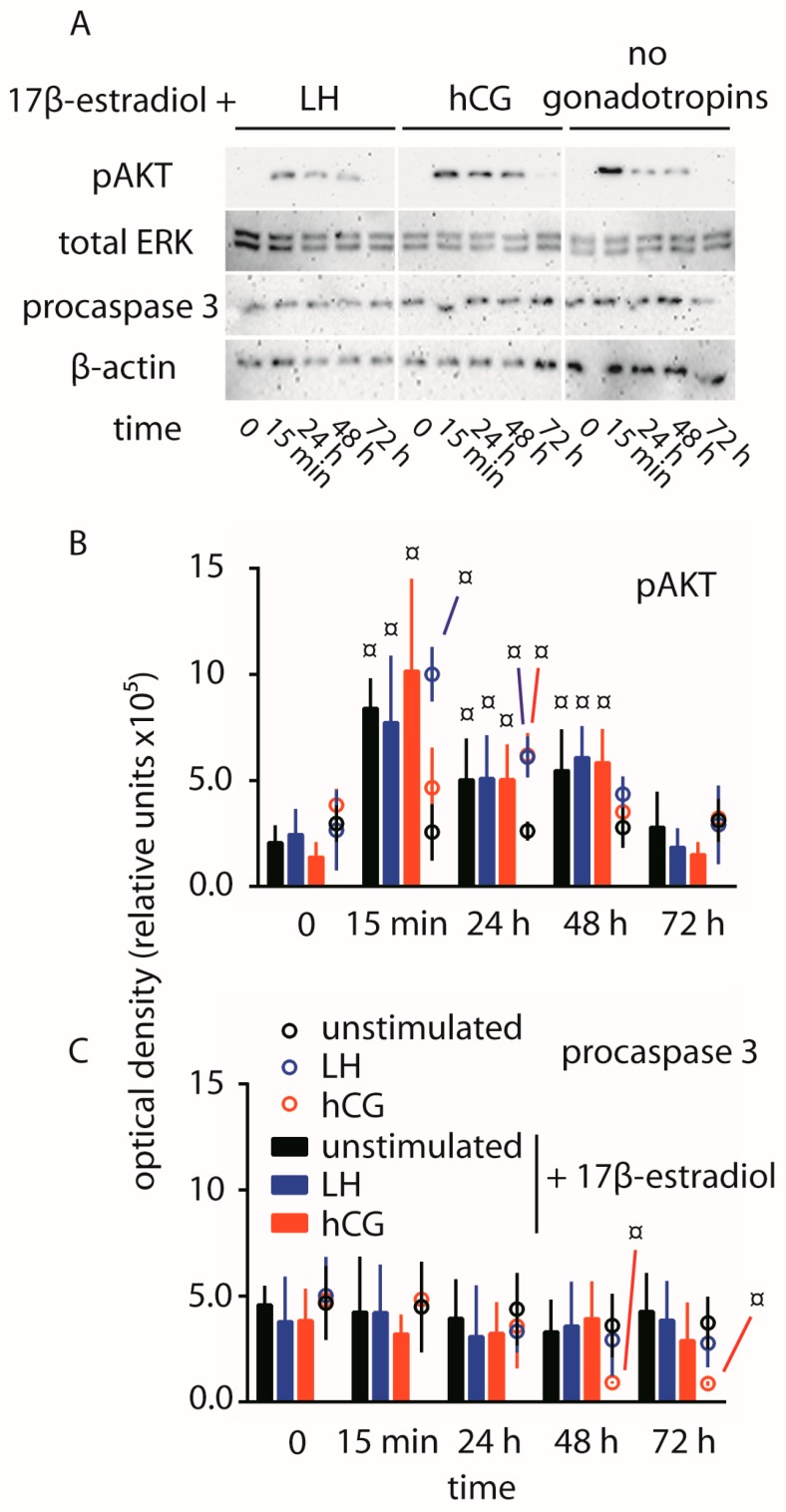
Comparison of pAKT activation and procaspase 3 cleavage over 72 h, in hGLC maintained under LH- or hCG-treatment in the presence of 17β-estradiol. (**A**) Evaluation of pAKT and procaspase 3 by Western blotting in cells treated 0–3 days by LH or hCG together with the estrogen. Unstimulated cells were maintained in the presence of 17β-estradiol too. Total ERK and β-actin served as loading controls (images representative of three independent experiments); (**B**,**C**) Semi-quantification of pAKT (**B**) and procaspase 3 (**C**) Western blotting signals. Data from samples maintained in the presence of 17β-estradiol are represented by bars, while circles were samples in the absence of the estrogens extracted from Figure 1 and Figure 2. ¤ = significantly different to unstimulated (control) at the time-point 0; two-way ANOVA and Bonferroni post-test (*p* < 0.05; means ± SD; *n* = 3).

Significantly higher pAKT activation occurs in the presence of 17β-estradiol within the time range 15 min–48 h, compared to controls (time-point 0), not depending on LH and hCG treatment (*t*-test; *p* < 0.05; *n* = 3) (Figure 5). Maximal level of phosphorylation was achieved within 15 min of exposure to 17β-estradiol, although the activation is detected over 48 h. Moreover, 17β-estradiol treatment results in not detectable procaspase 3 cleavage by Western blotting, also in the presence of LH or hCG.

**Figure 6:**
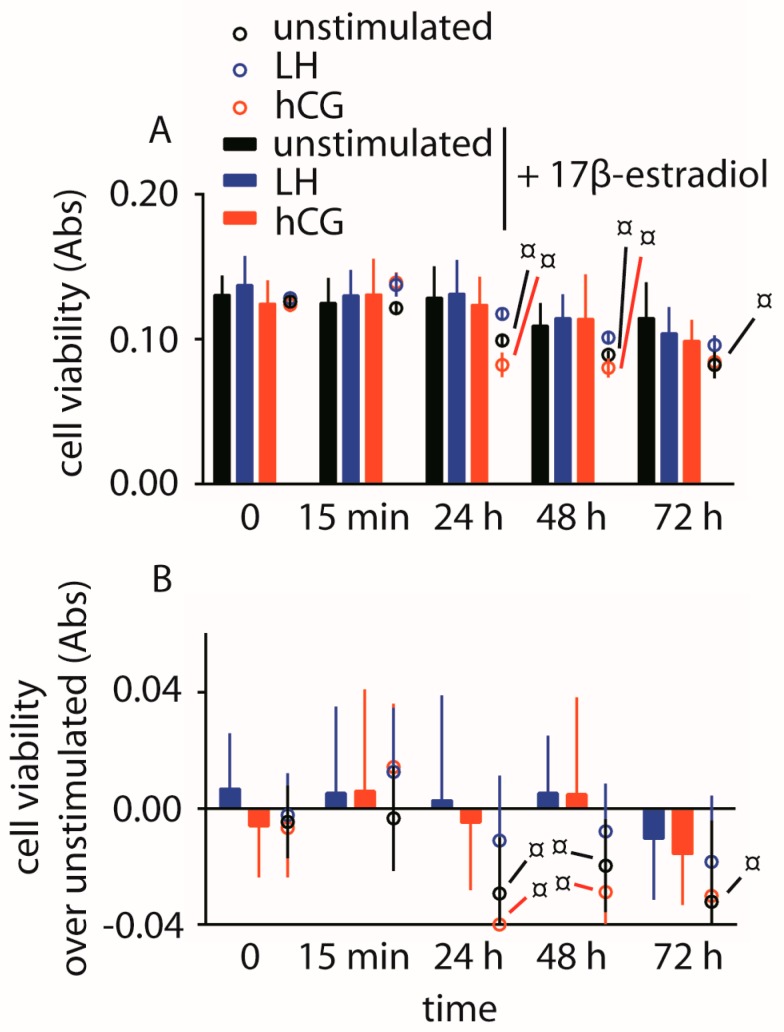
Analysis of hGLC viability by the MTT assay. Cells were maintained under LH or hCG treatment in the presence of 17β-estradiol. Cells maintained in the absence of gonadotropin served as controls. Data from samples maintained in the presence of 17β-estradiol are represented by bars, while circles were samples in the absence of the estrogens extracted from Figure 1 and Figure 2. (**A**) Directly measured absorbance values; (**B**) Cell viability values expressed as increase or decrease over basal levels. ¤ = significantly different versus same gonadotropin stimulation, at the same time-point; (two-way ANOVA and Bonferroni post-test; *p* < 0.05; means ± SD; *n* = 10).

The analysis of cell viability revealed similar results among 17β-estradiol-treated hGLC, not providing significantly different absorbance values between LH or hCG co-treatment (Figure 6).

## 3. Discussion

This study demonstrated that treatments by LH and hCG result in different effects on viability of both hGLC and hGL5/LHCGR cells. In particular, serum deprivation-induced pro-apoptotic stimuli were blocked by 100 pM LH, which rescued cells from apoptosis. LH treatment results in relatively high levels of ERK1/2 and, especially, AKT phosphorylation, anti-apoptotic *XIAP* gene expression and increased cell viability over three days. Cell treatment by hCG results instead in prevalent PKA-pathway activation, revealed by higher pCREB and *STARD1* gene expression levels than that obtained upon LH treatment. Procaspase 3 cleavage and decreased cell viability were also positively regulated by hCG treatment over three days. Interestingly, in serum starved cells, pro-apoptotic stimuli linked to hCG treatment were reversed by 17β-estradiol, which induced pAKT activation and blockade of procaspase 3 cleavage.

### 3.1. Gonadotropin-Mediated Intracellular Signaling In Vitro

In hGLC, previous studies demonstrated a different action of LH and hCG at the molecular level in vitro [14,16], revealing prevalent pERK1/2 and pAKT activation upon LH treatment, while intracellular cAMP achieved higher levels by hCG, which, therefore, possesses a higher steroidogenic potency. These gonadotropin-specific effects were amplified in the presence of FSH [16], suggesting that different activity at the receptor level and intracellular signaling activation characterize the two hormones [30]. Indeed, different binding affinity of LH and hCG for the rodent LH receptor was demonstrated three decades ago [31], providing the first evidence that these hormones interact with different binding sites of the common receptor. However, recent experiments demonstrated that only quantitatively, but not qualitatively different intracellular signaling is mediated by the rodent receptor, which resulted in prevalent cAMP/PKA-pathway activation induced more effectively by hCG than LH [18]. The qualitative discrimination between LH- and hCG-specific signaling may be due to the hinge region of the human receptor, which is encoded by a DNA sequence corresponding to exon 10 of the *LHCGR* gene [3], sharing only ~80% of identity with the rat and mouse LHR [18]. The importance of the amino acid sequence encoded by exon 10 is provided by the example of a *LHCGR* deletion involving the whole exon 10 found in a male patient affected by hypogonadism [32]. In this patient, only the administration of exogenous hCG, but not endogenous LH, restored the production of testosterone, suggesting that *LHCGR* exon 10 encodes for a crucial region in discriminating LH/hCG. Interestingly, the New World monkey *Callythrix jacchus* naturally lacks the LHCGR exon 10 and displays pituitary hCG-like, *LHB* mRNAs [33].

LH-dependent intracellular signaling is prevalently focused on ERK1/2- and AKT-pathway activation, rather than cAMP/PKA [14], resulting in the upregulation of proliferative and anti-apoptotic signals [16]. These results were confirmed in goat ovarian granulosa cells, in which long-term exposure to LH was linked to sustained cell proliferation, oppositely to what was observed using hCG [17]. On the other hand, hCG treatment led to relatively high levels of intracellular cAMP [17]. The convergence of results describing the action of hCG on cAMP/PKA-pathway, obtained in granulosa cells from human and goats, suggests that this hormone possesses higher steroidogenic potential than LH. However, studies evaluating specific LH- and hCG-mediated progesterone production failed to demonstrate any difference in granulosa cells [14,17], at least in the absence of FSH [16], suggesting that a number of intracellular factors, not yet identified, may play a role in the modulation of steroid synthesis. However, the two gonadotropins impact differently on intracellular events downstream the receptor-mediated steroidogenic signals, such as *STARD1* and *CYP19A1* gene expression. In our study, the higher *STARD1* gene expression levels, achieved upon hCG than LH treatment, suggest that the two hormones have different potency in activating molecular mechanisms regulating progesterone synthesis. Instead, we found higher levels of *CYP19A1* gene expression upon 72 h-LH than -hCG treatment. Aromatase enzyme is encoded by *CYP19A1* gene and serves to catalyze the conversion of androgen to estrogens in granulosa cells resulting in estradiol production, which is required to sustain folliculogenesis [5], thus reflecting the endocrine functions of LH at this stage.

### 3.2. Cross-Talk between Life and Death Signals

Number of researches described the relationship between the simultaneous activation of steroidogenic and pro-apoptotic pathways in granulosa cells [34,35,36,37]. It was proposed that gonadotropin-mediated apoptotic signals regulating follicular atresia occur bypassing the mitochondria, ensuring the onset and prosecution of steroidogenesis mediated by StAR and other enzymes [35]. In granulosa cells, death signals are linked to cAMP intracellular increase and were mimicked by forskolin or 8-bromo-cAMP treatment, which induced the activation of p53 and apoptosis [11], at least in vitro [13]. Since hCG is more potent than LH in inducing cAMP activation in hGLC [14], it is not surprising that CREB phosphorylation was higher upon hCG than LH treatment, confirming the preferential upregulation of the cAMP/PKA-pathway by hCG. However, this positive impact on the steroidogenic pathway may be linked to pro-apoptotic stimuli in long-term, serum starved hGLC in vitro, as our data and previous studies demonstrated [11,13,16,17]. The pro-apoptotic signals mediated by gonadotropins featured by relatively high steroidogenic potential was confirmed in both untransfected and FSHR-transfected hGL5 cells [9], where procaspase 3 cleavage was enhanced upon over-activation of the cAMP/PKA-pathway. Interestingly, while active caspase 3 was found at the protein level, no increased *CASP3* gene expression was found, suggesting that the transcriptional efficiency of this gene is not related to the protein activity, at least within the time-window considered.

The relationship between gonadotropin-mediated cAMP/PKA-pathway and pro-apoptotic stimuli in granulosa cells is still not completely clear and a number of opposite results were provided [38,39,40], suggesting that the role of the second messenger in determining the cell fate is depending on the type and metabolic state of the cells and may be tissue-specific in vivo. These considerations are consistent with the biphasic response to increasing FSH-induced cAMP concentrations observed in a pre-ovulatory, immortalized granulosa cell line (HGP53), where anti- or pro-apoptotic effects occurred at lower or higher cAMP intracellular levels [37]. In contrast, the positive effect of LH on cell viability [16,41] may be linked to the preferential activation of ERK1/2- and AKT-pathway compared to relatively low potency in inducing cAMP/PKA-pathway activation [14]. In this case, pERK1/2 activation may reduce cAMP/PKA-mediated pro-apoptotic signaling and gonadotropin desensitization, resulting in a lower rate of cell death [42], besides the well-known anti-apoptotic role exerted by AKT in granulosa cells [38]. Moreover, in vitro systems can not provide the whole picture of intracellular actions mediated by a wide number of cell signaling activators occurring in vivo. The abolition of hCG-dependent, pro-apoptotic signals by co-treatment with a physiological dose of 17β-estradiol supports this concept.

Interestingly, 15 min-treatment by hormones increased absorbance values evaluated by MTT assay (Figure 4). Since MTT is reduced to formazan by the dehydrogenase enzyme using succinate, reduced nicotinamide adenine dinucleotide (NADH) and nicotinamide adenine dinucleotide phosphate (NADPH) as substrate [43], we hypothesize that the 15 min-increased signals might be due to the rapid non-genomic action of the hormones on mitochondria. In fact, gonadotropins may impact on mitochondrial reduction via a rapid cAMP-dependent intracellular mechanism involving NADPH [44].

### 3.3. Estradiol as a Mediator of Proliferative and Anti-Apoptotic Effects

Since the 17,20 lyase activity of 17β-OH-dehydrogenase is lacking in hGLC, the steroidogenic pathway of these cells in vitro results in production of 17-(OH)-progesterone, which is not further converted to androgen. Therefore, these cells are unable to synthesize estradiol without the addition of androstenedione, normally provided by theca cells in vivo [5], as an androgenic substrate for aromatase in vitro. In this experimental context, the proliferative effect of estrogens was exerted by addition of ~200 pg 17β-estradiol to the cell media, resulting in gonadotropin-independent AKT phosphorylation and prevention of procaspase 3 cleavage. This is consistent with the proliferative/anti-apoptotic role of estrogens [45,46], which may rescue hGLC from serum-starved, hCG-induced cell death in vitro. Life signals are induced by estradiol via AKT-pathway through the nuclear receptor and a membrane GPCR, resulting in the regulation of ovarian functions [47], trophoblast invasion [48], cell tumor growth [49], etc. The experimental evidence that pro-apoptotic effects mediated by gonadotropins are blocked by estradiol may explain why high doses of exogenous gonadotropins used for ART, i.e., FSH and hCG, induce multi-follicular ovulation instead of atresia in vivo.

## 4. Materials and Methods

### 4.1. Recombinant Gonadotropins

Human recombinant LH (Luveris) and hCG (Ovitrelle) were provided by Merck KGaA (Darmstadt, Germany).

### 4.2. Patients’ Selection

Patients undergoing oocyte retrieval for assisted reproduction at the Unit of Obstetrics and Gynecology of the IRCCS-Arcispedale Santa Maria Nuova (Reggio Emilia, Italy) provided the hGLC used in this study. These patients are infertile due to tubal or male factor and were treated as previously described [16]. Patients had to match the following criteria: age of 25–45 years, absence of endocrine abnormalities and severe viral or bacterial infections. hGLC were collected under the permission of the local Ethics Committee and written consent signed by patients (disposizione n. 796, 19 June 2014, released by Comitato Etico Provinciale, Reggio Emilia, Italy).

### 4.3. Granulosa Cells Isolation and Culture

hGLC consist of waste products from the clinical procedure of oocyte retrieval for assisted reproduction. Cells were purified by 50% Percoll density gradient (GE Healthcare, Little Chalfont, UK) using a method previously described [14,16,28,50]. Cells from 3–4 patients were pooled, seeded in multi-well plates and maintained in McCoy’s 5 A medium (Gibco, Thermo-Fisher Scientific, Waltham, MA, USA), supplemented with 5% fetal bovine serum, 250 ng/mL Fungizone (all from Sigma-Aldrich, Saint Louis, MO, USA), 100 U/mL penicillin, 50 µg/mL streptomycin and 25 mM Hepes (all from Gibco). Cells were maintained five days in an incubator at 37 °C and 5% CO_2_ to recover gonadotropin receptor gene expression [50] and serum-starved over-night before stimulation. The five days-period of cell culture is required to treat the cells by gonadotropins and perform in vitro analyses, which are not affected by the stimulation protocol of the donor. Moreover, all the stimulations were performed in the absence of serum to induce pro-apoptotic stimuli and to avoid background signals [16,20].

### 4.4. Cell Line

A immortalized human primary granulosa (hGL5) cell line [51] was used for control experiments. hGL5 cells permanently overexpressing LHCGR (hGL5/LHCGR) were developed and characterized previously [9,14]. Cell line were maintained in an incubator at 37 °C and 5% CO_2_, in DMEM/F12 medium supplemented with 10% fetal bovine serum (Sigma-Aldrich), 100 U/mL penicillin, 50 µg/mL streptomycin (all from Gibco) and 2% ITS + Premix Universal Culture Supplement (#354352; Corning Incorporated, Corning, NY, USA).

### 4.5. Western Blot Analysis and Antibodies

As indication of the activation of proliferative, anti-apoptotic and steroidogenic pathways, we analyzed pERK1/2, pAKT and pCREB levels, respectively, which were induced by LH or hCG treatment by Western blotting, as previously described [9,14,16,18,28]. Briefly, cells were seeded in 24-well plates (1 × 10^5^ cells/well) and maintained under continuous treatment with 100 pM LH or hCG, as a gonadotropin dose maximally activating the above signaling pathways in hGLC [9,14,16,28]. Furthermore, 730 pM 17β-estradiol (~200 pg/mL) was also used where appropriated. Cells were lysed at specific time-points (0–72 h range) for protein extraction in 4 °C RIPA buffer added with PhosStop phosphatase inhibitor cocktail and protease inhibitor cocktail (Roche, Basel, Switzerland). pERK1/2, pAKT and pCREB activation were evaluated by 12% SDS-PAGE and Western blotting, using specific antibodies (#9101, #9271 and #9198, respectively; Cell Signaling Technology Inc., Danvers, MA, USA). Monoclonal caspase 3 (active/pro) antibody (#MA1-91637; Thermo-Fisher Scientific) was used. Total ERK1/2 (#4695; Cell Signaling Technology Inc.) and horseradish peroxidase (HRP)-conjugated β-actin antibody (#A3854; Sigma-Aldrich) served as loading control where appropriate. Signals were revealed by ECL chemiluminescent compound (GE HealthCare), after incubation of the membranes with a secondary anti-rabbit HRP-conjugated antibody (#NA9340V; GE HealthCare), except for β-actin. Western blotting signals were acquired and semi-quantitatively evaluated by the QuantityOne analysis software (Bio-Rad Laboratories Inc., Hercules, CA, USA) and then represented as means ± SD.

### 4.6. Stimulation and Evaluation of Gene Expression

For the experiments, 100 pM LH and hCG dose maximally activating cell signaling [9,14,16,28] was used for gene expression analysis. Cells were seeded in 24-well plates (1 × 10^5^ cells/well) and maintained under continuous stimulation by LH and hCG. Gonadotropins are highly stable in culture media and are measurable after several hours, as previously observed [14]. Samples were lysed and subjected to RNA extraction using the automated extractor EZ1 Advanced XL (Qiagen, Hilden, Germany). Equal amounts of total RNA were retro-transcribed by iScript reverse transcriptase (Bio-Rad Laboratories Inc.) according to the following protocol, as previously described [18]: 25 °C for 5 min; 42 °C for 30 min; 85 °C for 5 min. Quantitative real-time PCR was performed in triplicates using specific gene primer sequences (Table 1) and settings: 95.0 °C for 30 s; 95.0 °C for 3 s and 45 cycles; 57.0 °C for 30 s. Normalized gene over the *ribosomal protein S7* (*RPS7*) gene expression was evaluated using the 2^-ΔΔ*C*t^ method [52]. Real-time PCR method and primer sequences were validated previously [14,16,28].

### 4.7. Analysis of Cell Viability

Cells were seeded at in 96-well plates (1 × 10^4^ cells/well) and maintained under continuous stimulation by 100 pM LH or hCG. 730 pM 17β-estradiol was also used where appropriated. The viability of hGLC and hGL5/LHCGR cell line was assessed by MTT assay [53]. The absorbance was detected by a Victor3 plate reader (Perkin Elmer Inc., Waltham, MA, USA) and represented as means ± SD in a graph, as a measure of cell viability.

### 4.8. Statistical Analysis

Data were plotted as means ± SD and normalized over control as indicated. Data from Western blotting signals and gene expression analysis were represented as relative units and relative expression, respectively. *t*-Test or two-way ANOVA followed by Bonferroni post-test was performed as appropriate. Differences were considered significant for *p* < 0.05. Statistical analysis was performed using the GraphPad Prism software v 6.01 (GraphPad Software Inc., San Diego, CA, USA).

## 5. Conclusions

Gonadotropins mediate life and death signals in vitro through a fine-tuned regulation of different signaling pathways simultaneously activated. The proliferative role of LH exerted during folliculogenesis is reflected by the activation of ERK1/2- and AKT-pathways, anti-apoptotic gene expression and increased granulosa cell viability. hCG is the pregnancy hormone displaying relatively high steroidogenic potential mediated via signaling pathways linked to apoptotic signals in vitro, indeed resulting in procaspase 3 cleavage and decreased cell viability. This is apparently in contrast with what is observed in vivo during hCG supplementation in controlled ovarian stimulation. Here, we show that the hGLC response to LH and hCG is modulated by estrogens, which may counteract the pro-apoptotic, hCG-mediated signal. The mechanism thereof remains to be explored.

## Figures and Tables

**Table 1:** List of the primer sequences used for real time PCR analysis. National Center for Biotechnology Information (NCBI) or GenBank accession numbers are also provided.

Gene and Primer Type	Primer Sequence from 5′ to 3′	NCBI or GenBank Accession Number
*CCND2* forward	CTGGCCTCCAAACTCAAAGA	NM_001759.3
*CCND2* reverse	TTCCACTTCAACTTCCCCA	
*CASP3* forward	TGTTTGTGTGCTTCTGAGCC	AY219866.1
*CASP3* reverse	TCTACAACGATCCCCTCTGAA	
*TP53* forward	GAGAACACCGCTTGGAACTA	NG_017013.2
*TP53* reverse	AGCCCACTTACAGCCTTTCA	
*XIAP* forward	TTGAAAATAGTGCCACGCAG	NM_001167.3
*XIAP* reverse	TGTGTCTCAGATGGCCTGTC	
*STARD1* forward	AAGAGGGCTGGAAGAAGGAG	NM_000349.2
*STARD1* reverse	TCTCCTTGACATTGGGGTTC	
*CYP19A1* forward	CCCTTCTGCGTCGTGTCAT	NM_000103.3
*CYP19A1* reverse	GATTTTAACCACGATAGCACTTTCG	
*RPS7* forward	AATCTTTGTTCCCGTTCCTCA	NM_001011.3
*RPS7* reverse	TTCTGCCTAAGCCAACTCG	

## Supplementary Materials

Supplementary materials can be found at www.mdpi.com/1422-0067/18/5/926/s1.
