# Supplementary material for: Estrogen Modulates Specific Life and Death Signals Induced by LH and hCG in Human Primary Granulosa Cells In Vitro
Source: Int J Mol Sci. 2017 Apr 28;18(5):926. doi: 10.3390/ijms18050926 (PMC5454839; doi:10.3390/ijms18050926)
Supplement: Supplementary file 1 [file ijms-18-00926-s001.pdf]

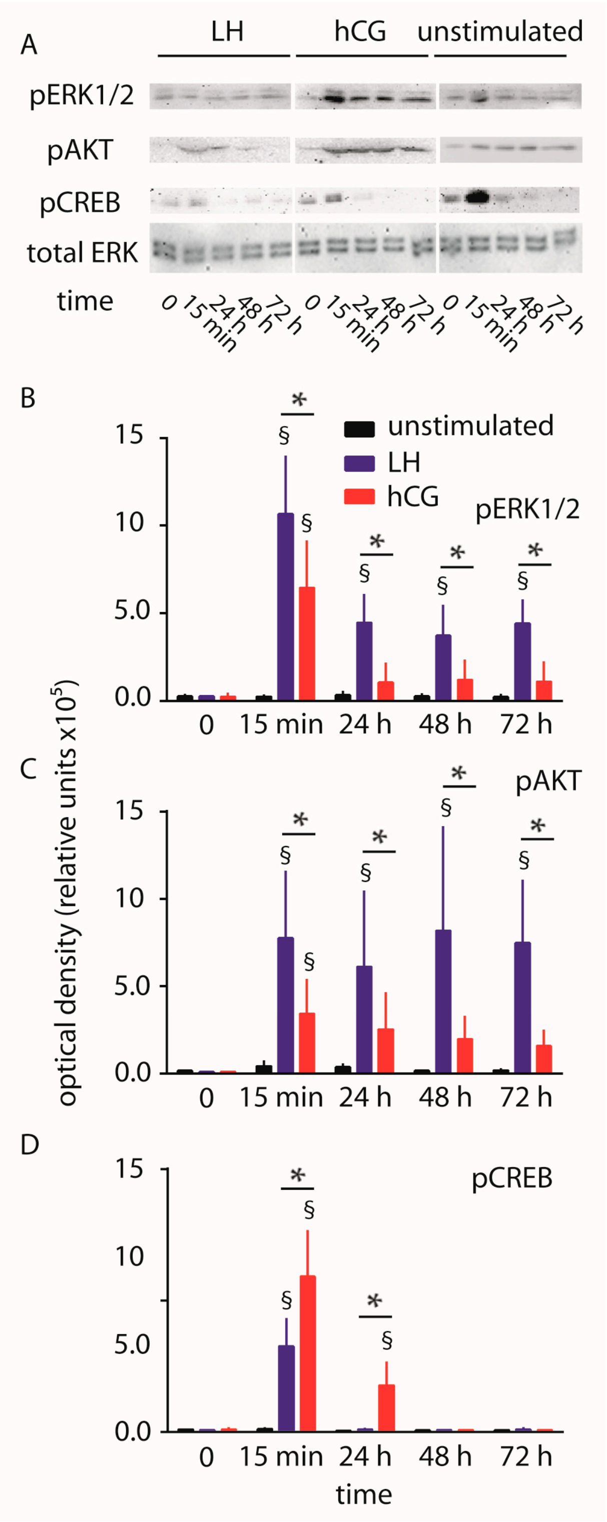

Figure S1. Comparison of LH- and hCG-induced ERK1/2, AKT and CREB phosphorylation over 72 h, in the hGL5/LHCGR cell line. A) Evaluation of pERK1/2, pAKT and pCREB activation by Western blotting. Total ERK served as loading control (images representative of four independent experiments). B-D) Semi-quantification of pERK1/2, pAKT and pCREB Western blotting signals. §=significantly different to unstimulated (control) at the same time-point; *=significant difference of LH versus hCG; two-way Anova and Bonferroni post-test ($p<0.05$; means ± SD; n=4).

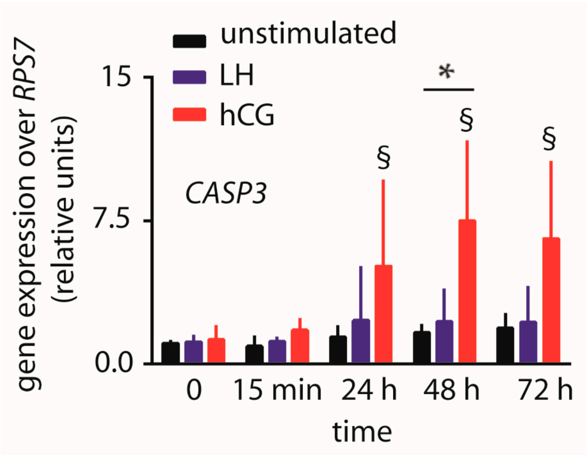

Figure S2. Time-course (0-72 h) analysis of LH- and hCG-induced *CASP3* gene expression, by real time PCR. Values were normalized over the expression of RPS7 housekeeping gene. §=significantly different to unstimulated (control) at the same time-point; *=significant difference of LH versus hCG; two-way Anova and Bonferroni post-test ($p<0.05$; means ± SD; n=3).

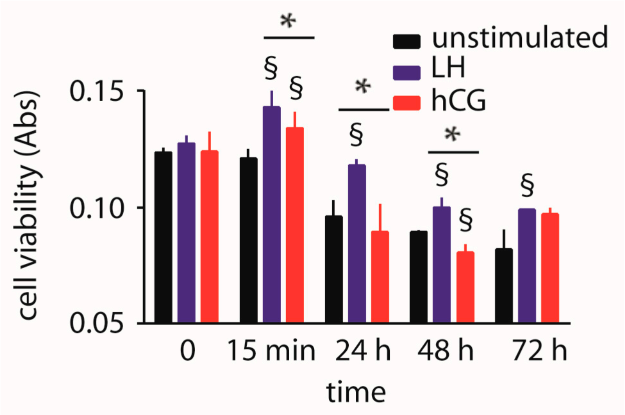

Figure S3. Analysis of LH- and hCG-treated (0-72 h), serum-starved hGL5/LHCGR cell viability, by MTT assay. Cells maintained in the absence of gonadotropin served as controls. §=significantly different to unstimulated (control) at the same time-point; *=significant difference of LH versus hCG; two-way Anova and Bonferroni post-test ($p<0.05$; means ± SD; means ± SD; n=10).
